# Analyzing the Spatio-Temporal Dynamics of Urban Land Use Expansion and Its Influencing Factors in Zhejiang Province, China

**DOI:** 10.3390/ijerph192416580

**Published:** 2022-12-09

**Authors:** Yue Wu, Zexu Han, Auwalu Faisal Koko, Siyuan Zhang, Nan Ding, Jiayang Luo

**Affiliations:** 1 College of Civil Engineering and Architecture, Zhejiang University, Hangzhou 310058, China; 2 International Center for Architecture and Urban Development Studies, Zhejiang University, Hangzhou 310058, China

**Keywords:** urbanization, urban land use, urban expansion, GIS, spatial Durbin model, Zhejiang province

## Abstract

The 21st century expansion of built-up areas due to rapid urbanization has recently been at the forefront of global land use/land cover research. Knowledge of the changing dynamics of urban land use is crucial for the monitoring of urbanization and the promotion of sustainable urban development. In this paper, Zhejiang Province was selected as the study area. It is a region with rapid urban growth located along the southeastern coast of China, with a highly developed economy but with a shortage of land resources. We employed remotely sensed and socio-economic panel data for the period between 1990 and 2020 to monitor urban land use changes and utilized the spatial Durbin model (SDM) to examine the urbanization process and the various driving factors of rapid urban expansion in Zhejiang Province, China, from 1990 to 2020. The study’s results revealed substantial urban growth of about 6899.59 km^2^, i.e., 6.6%, whereas agricultural land decreased by 4320.68 km^2^, i.e., 4.19%. The rapid urban development was primarily attributed to the transformation of farmlands, forestlands, and water bodies into built-up areas by nearly 86.9%, 6.94%, and 6.06%, respectively. The built-up areas revealed features of spatial clustering. The study showed that the expansion hotspots were mainly distributed within the urban fabric of cities such as Hangzhou, Ningbo, Jinhua–Yiwu, and Wenzhou–Taizhou. The results further revealed the substantial influence of urban growth on the local areas of the province. As the core explanatory variables, population and economic development significantly promoted local urban expansion. The study’s findings indicated a positive spatial spillover effect as regards the influence of economic development on the study area’s urban growth, whereas the spatial spillover effect of the population was negative. Therefore, economic development was a major driving factor contributing immensely to the expansion of urban areas in Zhejiang Province, especially in the 26 mountainous counties of the province. The study enriches our understanding of the transformation of LULC and the changing dynamics of urban areas in China and provides the necessary research data that are vital for urban land-use planners and decision-makers to overcome the negative consequences of the expansion of urban areas due to the continuous economic growth of China.

## 1. Introduction

Land is an indispensable natural resource that contributes to local, regional, and global economic development. The continuous alteration of land use/land cover (LULC) patterns due to socio-economic development has recently been at the forefront of global land use research. As the world’s most populous and the largest developing nation, China has achieved rapid, long-term sustained economic growth and large-scale urbanization since the implementation of the country’s reform and opening up in 1978 [1,2]. The urbanization rate in China has increased tremendously over the years, from 17.92% in 1978 to 63.89% in 2020, indicating exponential urban growth characteristics. Rapid urbanization has not only led to a strong impetus for the country’s continuous growth and socio-economic development but has contributed to the significant changes in the land use/land cover patterns of provinces due to the expansion and development of built-up areas [3,4,5]. The consequences of this urban growth are a series of environmental challenges that include but are not limited to the reduction of agricultural and forest land, which contributes to climate change. Meanwhile, ecological deterioration due to urban expansion results in a more prominent contradiction between land supply and demand [6,7,8].

Several researchers have recently studied the transformation of Land use/land cover (LULC) patterns at a regional and local level [9,10,11]. Such studies include China’s urban expansion and built-up areas at a national scale [11,12,13]. At different regional levels, LULC alterations have been examined in China in the areas of Beijing–Tianjin–Hebei [14], the Yangtze River [15,16,17], the Pearl River Delta [18,19], and large cities that include Beijing [20,21], Tianjin [22,23], Shanghai [24,25], Wuhan [26,27], and Hangzhou [28]. Similarly, Xu et al. [29] analyzed the changing dynamics of land use/land cover at the county level. The findings of these studies have provided relevant literature and scientific results that have enriched the understanding of the transformation of LULC and the changing dynamics of urban areas in China. These studies have also provided data that have helped to attain sustainable urban development in different cities in China. Recent studies conducted using different research approaches have attributed the expansion of built-up areas in most cities and urban centers to several influencing factors [30,31]. These factors are the drivers of urban development and include but are not limited to environmental factors [32,33,34]; population growth [16,35,36]; socio-economic development [37,38,39]; infrastructural facilities [40,41]; openness, i.e., accessibility to regions of the world [42]; and others [43].

Remote sensing data and various geospatial techniques have been used in previous studies to establish spatial analysis models to monitor and analyze urban land uses in different cities and regions [44,45]. Analytical approaches that utilize statistical data and adopt techniques such as traditional econometric regression analysis, correlation analysis, and principal component analysis have been conducted in empirical studies of different scales to determine the influence of various socio-economic factors on urban land use changes [46,47,48]. However, the existing literature exhibits a significant limitation in studies of LULC. LULC data for previous periods are discontinuous. In addition, the adjourning neighboring areas can positively or negatively influence the evolution of the urban fabric of a city or region. Urban land use transformations may have spatial spillover effects due to various drivers of urban expansion. Many studies have often ignored the indirect influence of these urban expansion drivers on the adjoining neighboring areas. Therefore, the spatio-temporal dynamics of urban land use and the utilization of spatial econometric methods are necessary for considering the spatial influence of economic models in the expansion of urban areas [49]. As early as 2003, Overmars et al. [50] suggested that due to the potential of spatial autocorrelation in LULC studies, it was vital to conduct spatial autocorrelation analysis first and then introduce a spatial econometric model for the analysis of the results. Several researchers have employed similar methods in some regions of China; however, this method has not been applied to Zhejiang Province. Wang analyzed the direct impact and spillover effect of regional economic development on the expansion of built-up areas in China [51]. That study provided an empirical basis for controlling the irrational expansion of China’s urban areas. Chen et al. [52] analyzed the driving mechanism of urban-rural development based on provincial panel data and put forward countermeasures and suggestions for the differentiated development of China’s eastern, central, and western regions. Tang et al. [53] utilized the spatial lag model, the spatial error model and the spatial Durbin model to analyze the relationship between urban land and regional economic development at the county level in the Beijing–Tianjin–Hebei region. Furthermore, the spatial dependence of the mechanism behind the expansion of urban land was previously studied in China [54,55]. However, most of these studies have continued to use spatial lag or spatial error models, whereas the spatial Durbin model is rarely utilized. The spatial lag or spatial error model only considers the spatial autocorrelation of the dependent variable, i.e., the error term, and does not consider the spatial correlation of bivariate and multivariate variables.

Therefore, in the present study, we employed remotely sensed images obtained at uniform time intervals to build a spatial Durbin model using Zhejiang Province on China’s eastern coast as the study area. The model incorporated the geographical spatial connections between elements using various geospatial techniques to analyze the change dynamics of urban land uses from 1990 to 2020 and the various drivers of these LULC changes. The findings of this study will provide the necessary research data that are crucial for the effective planning of urban development and the management of natural land resources.

## 2. Study Area and Data Sources

### 2.1. The Study Area

**Figure 1:**
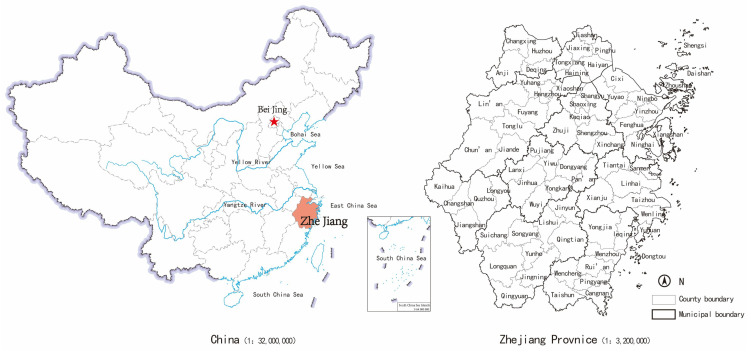
Location of Zhejiang Province in China.

Zhejiang Province is located on the southern boundary of the Yangtze River Delta along the South-Eastern coast of China. The province shares a border with the East China Sea and covers a landmass of approximately 105,000 square kilometers, as shown in Figure 1.

Zhejiang is one of the provinces with the fastest economic growth rate and is among the most vibrant regions in China. In 2020, the region’s GDP reached 6.46 trillion yuan, equivalent to 100 times that of 1978 based on comparable national data. The region’s digital economy and green development are the two most recognizable factors that have contributed to the growth of GDP in Zhejiang Province and China as a whole. Zhejiang Province is also one of the regions with the most balanced development of urban and rural areas in China. In 2020, the province’s population of permanent urban dwellers was approximately 65 million, with an urbanization rate of about 72.2%. The ratio of income between urban and rural residents was 1.96:1, far below China’s national average level of 2.1:1. In 2021, Zhejiang was designated for the vital task of building a “demonstration zone for common prosperity” and was chosen as a study area for the exploration of ways to achieve shared prosperity in China.

However, in recent years, Zhejiang province has faced a series of challenges that include a shortage of land resources and a weak environmental carrying capacity. According to China’s third national land survey, by the end of 2019, the per capita area of cultivated land in Zhejiang was 0.30 mu. This indicates that the province accommodates only 22.05% of the national per capita, i.e., 1.36 mu.

This output is far lower than the international per capita cultivated land warning line of 0.795 mu, determined by the Food and Agriculture Organization (FAO) of the United Nations. In the past 40 years of China’s reform and opening up process, which began in 1978, the large-scale expansion of urban land use caused by rapid urbanization has aggravated the shortage of land resources and led to problems related to supply and demand in most provinces in China, especially in Zhejiang Province.

### 2.2. Sources of Data

Remote sensing data related to LULC from 1990 to 2020 were acquired at equal intervals from the annual Classified Land Cover Products (CLCD) of China published in the Zenodo database. Jie Yang and Xin Huang of Wuhan University, China, obtained the satellite data using the Google Earth Engine and the random forest classification method to interpret 335,709 Landsat satellite images [12]. The satellite images had a spatial resolution of 30 m, and the overall accuracy of the CLCD was 79.31%. Based on the evaluation of 5131 third-party test samples, it was found that the overall accuracy of the CLCD data was better than that of the current mainstream data products such as MCD12Q1, ESACCI_LC, FROM_GLC, and GlobeLand30. In addition, the comparison of CLCD with several Landsat-derived thematic products also showed good agreement with global forest change, global surface water, and three impervious surface products [12].

The socio-economic data for the study area were retrieved from the annual statistical data of Zhejiang Province that have been published over the years, whereas data that were not available for the province were obtained from the statistical data in China’s national economic and social development reports based on counties and cities. In the statistical yearbook, the data for the central urban areas of some cities are often published together. For example, there are no separate data for Shangcheng District, Xiacheng District, West Lake District, Gongshu District, Jianggan District, and Binjiang District of Hangzhou. The data for the Hangzhou urban area are used as the total data for the above six districts. We also followed this classification method in our analysis, and finally 90 counties were merged into 72.

### 2.3. Variable Selection

The major factors influencing the expansion of urban land use can be categorized as natural factors and socio-economic factors [56]. Natural environmental factors mainly affect the direction, speed, and mode of urban expansion through the interaction of climate, hydrology, soil, topography, and other environmental factors. However, this type of factor has a long-term cumulative effect that is not visible in the short term, compared to the influence of socio-economic factors, which have a short-term impact. Socio-economic factors such as population and economic growth have contributed to the undesirable challenges of rapid urban expansion experienced in most urban centers.

Based on a review of the relevant literature, in our study we comprehensively considered the actual situation of socio-economic development in the study area, i.e., Zhejiang Province, and selected the region’s total population (RTP) and the gross domestic product (GDP) as the main explanatory variables based on the officially released statistical data for the province and country. Industrial structure (STR), foreign direct investment (FDI), fixed-asset investment (INV), and local fiscal expenditure (LFE) were the study’s controlled variables presented in Table 1. Population growth has been identified as a direct driving force for the expansion of built-up urban areas in the cities of developing nations [57]. The demand for land to meet people’s needs for development by various sectors of the economy essentially reflects the demand for land by the population. Land accommodates economic activities, and the development of built-up areas is an intuitive expression of economic growth. Different sectors of the economy have different needs for land resources. Compared with industrial sectors in secondary enterprises, institutions from tertiary sectors, such as educational and cultural facilities, are mainly located in urban centers with a higher floor area ratio and occupy relatively less built-up land [53]. Fixed-asset investment is mainly reflected in infrastructure construction and real estate development, which directly reflects the pace of urban development and foreign investment [58]. The influence of economic globalization has gradually deepened since the 1990s. The outlook regarding global land use and regional economics mainly changes through foreign direct investment, which creates multiple challenges for the urbanization of host countries, with an increasing characteristic of openness [59,60]. Other local fiscal expenditures, which include infrastructure investment, administrative expenditure, and many others, may also directly impact cities through an expansion in urban land use.

## 3. Research Methodology

### 3.1. Framework of the Study

**Figure 2:**
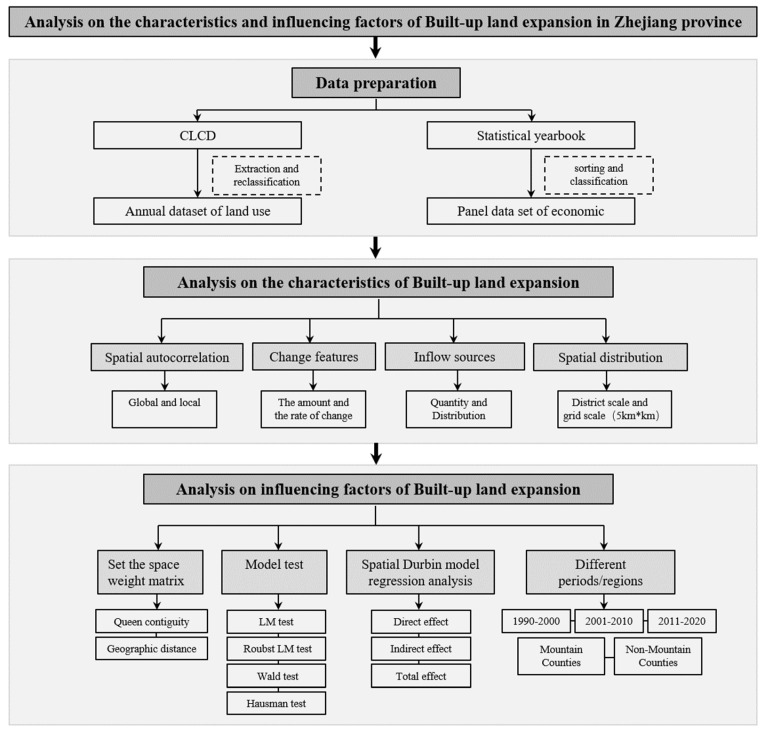
Structure of the research.

A methodological flowchart for this study is presented in Figure 2. The CLCD data were divided into nine types of land use, namely, cropland, forest, shrubland, grassland, water, snow/ice, barren, impervious, and wetland. According to the actual situation of Zhejiang Province, in our study we reclassified LULC into six categories, namely, built-up land (BL), farmland (FL), forest areas (FO), grassland (GR), water bodies (WB), and bare soil (SL). Among these, forest and shrublands were merged into forest areas, and water and wetlands were merged into water bodies. In addition, there was no snow/ice-type land due to the subtropical monsoon climate of Zhejiang. Meanwhile, we divided Zhejiang Province into a grid of 100 m × 100 m, and finally formed an annual dataset reflecting land use in Zhejiang Province. At the same time, the statistical data of the study area from throughout the study period were obtained to form the economic and social panel dataset of each county in Zhejiang Province. We then produced a spatial weight matrix and performed a series of tests on the three common spatial econometric models to determine the final model to utilize in this study. Geospatial techniques were used to analyze the urban expansion and change dynamics of urban land uses in Zhejiang Province during the study period. In addition, we mapped the spatial changes and presented the transfer matrix of land uses. The classified land uses were correlated with the socio-economic panel data of each county of the province, and the spatial econometric model was used to analyze the various factors influencing the region’s urban expansion. Finally, we examined the differences between the results obtained for different periods and regions of Zhejiang province.

### 3.2. Space Weight Matrix

Establishing a spatial weight matrix formed the basis of this study’s subsequent spatial correlation tests and spatial econometric model analysis. The spatial weight matrix is the main tool used in such studies to represent space in an abstract way and reflect spatial influences [61,62]. In order to ensure the effectiveness and reliability of the subsequent regression analysis, we produced two types of matrixes, comprising the queen contiguity weight $W_{1}$ and the geographic distance weight $W_{2}$. The matrices were utilized for the regression analysis and the comparison of results in this study.

#### 3.2.1. Queen Contiguity Weight

The queen contiguity weight was one of the first-order contiguity weights proposed by Berry and Marble [63], which exhibit movement methods that are similar to those in the concept of chess. Two areas having a common edge or vertex are defined as neighbors, and the space weight matrix element is 1; otherwise, it is 0. The mathematical expression for the queen contiguity weight is shown in Equation (1) below:

(1) $$W_{1}=\left\{\begin{array}{c} 1,\;j\in N\left(i\right)\\ 0,\;j\notin N\left(i\right) \end{array}\right.$$

where $N\left(i\right)$ is the neighbor set of region *i*.

#### 3.2.2. Geographic Distance Weight

The first law of geography states that there is a general connection between objects, with closer objects being more closely related than distant ones [64]. It is therefore not suitable to select the spatial weight matrix solely based on geographical contiguity conditions without considering the influence of the spatial distance between regions. Most studies suggest that the strength of the spatial correlation effect depends on distance. Therefore, the greater the distance between two spatial units, the weaker the spatial correlation [65]. The inverse distance spatial weight matrix is established using Equation (2).

(2) $$W_{2}=\left\{\begin{array}{c} 1/d_{ij}^{\gamma },\;i\neq j\\ 0,\;i=j \end{array}\right.$$

where $d_{ij}$ represents the distance between the geometric center of region *i* and *j*, and γ is set as 1.

### 3.3. Spatial Autocorrelation Test

Spatial autocorrelation represents the systematic variation of the value of a variable in space, which is usually generated by the interdependence of spatial units. It is an essential characteristic of geospatial phenomena and spatial processes. As Goodchild et al. [66] suggested, almost all data are spatially dependent, and this spatial dependency makes traditional econometric analysis biased. The measurement and testing of spatial autocorrelation are, therefore, closely related to the spatial scope and scale of this study. Most spatial autocorrelation tests are analyzed using either global or local spatial autocorrelation.

#### 3.3.1. Global Spatial Autocorrelation

Moran’s I index was employed to analyze the global spatial autocorrelation performed in this study. It was calculated using the mathematical formula presented in Equation (3):

(3) $$I=\sum_{i=1}^{n}\sum_{j=1}^{n}w_{ij}\left(X_{i}-\bar{X}\right)\left(X_{j}-\bar{X}\right)/\left(S^{2}\sum_{i=1}^{n}\sum_{j=1}^{n}w_{ij}\right)$$

where *n* is the total number of counties, $w_{ij}$ is the spatial weight matrix, $X_{i}$ is the attribute value of the region *i*, $\bar{X}=\frac{1}{n}\sum_{i=1}^{n}X_{i}$, $\mathrm{and}\;S^{2}=\frac{1}{n}\sum_{i=1}^{n}{\left(X_{i}-\bar{X}\right)}^{2}$).

The value range of Moran’s I index is [−1, 1]; values greater than 0 indicate positive auto-correlation, with values closer to 1 indicating stronger positive auto-correlation. Values less than 0 signify a negative autocorrelation, with values closer to −1 indicating a strong negative autocorrelation. The closer the value is to 0, the greater the likelihood of a random distribution and the independency of observations in the geographic space.

#### 3.3.2. Local Spatial Autocorrelation

Local spatial autocorrelation is the spatial decomposition of the global spatial correlation measurement index. The LISA index that is often used for this measurement not only reflects the correlation characteristics of each spatial element with its surrounding neighbors but can also be used to identify “cluster areas”, i.e., high-value and low-value agglomeration areas and “hotspot areas”, i.e., an area with a completely different value from those of its surrounding neighbors. The formula for calculating the local spatial autocorrelation is presented in Equation (4):

(4) $$I_{i}=\sum_{j\neq i}^{n}\frac{W_{ij}\left(X_{i}-\bar{X}\right)\left(X_{j}-\bar{X}\right)}{S^{2}}$$

where $I_{i}$ represents the specific value of LISA, which may be positive or negative. Positive values represent areas with high values that surround neighboring high values, and low values surround neighboring low values. Negative values indicate areas where high values are surrounded by low neighboring values, and low values are surrounded by high neighboring values. Other symbols have the same meanings as above.

### 3.4. Spatial Econometric Model

When the variables are spatially autocorrelated, the spatial econometric model is more applicable. Classical spatial econometric models include the spatial lag model (SLM), the spatial error model (SEM), and the spatial Durbin model (SDM).

The main difference between the three models is that they reflect different forms of spatial correlation. The spatial correlation of the SLM is reflected in the dependent variables of the model. The spatial correlation of the SEM is reflected in the random error term of the model (i.e., unobservable influencing factors). The spatial correlation of SDM is not only reflected in the dependent variable, but also in the bivariate spatial relationship between the dependent variable and the independent variable.

The formula of the spatial lag model (SLM) is

(5) $$y=\rho Wy+X\beta +\varepsilon ,\;\varepsilon \sim N\left[0,\sigma^{2}I\right],$$

where *y* is the explained variable (BUA); ρ is the spatial autoregressive coefficient; *Wy* is the spatial lag term of explained variable; and *X* is an *NT*×*K* matrix of the explanatory variables, with *N*, *T* and *K* representing the number of counties, the research period, and the six potential driving factors (RTP, GDP, STR, FDI, INV, LFE), respectively. β is the regression coefficient, ε is the random error term, and *W* is a *N*×*N* spatial weight matrix.

The formula of the spatial error model (SEM) is

(6) $$y=X\beta +u,u=\lambda Wu+\varepsilon ,\varepsilon \sim N\left[0,\sigma^{2}I\right],$$

where *λ* is the spatial error coefficient, which represents the impact of the errors of neighboring areas on the local area, and Wu is the spatial lag term of the error term. Other symbols have the same meanings as above.

The formula of the spatial Durbin model (SDM) is

(7) $$y=\rho Wy+X\beta +WX\theta +\varepsilon ,\;\varepsilon \sim N\left[0,\sigma^{2}I\right],$$

where θ is a parameter vector, representing the immediate marginal impact of the explanatory variables of neighboring areas on the explained variables. Other symbols have the same meanings as above.

## 4. Results

### 4.1. Outcome of the Spatial Autocorrelation

#### 4.1.1. Global Spatial Autocorrelation Results

To analyze the global autocorrelation for the study period between 1990 and 2020, we determined and utilized the built-up land area of each district and county in the study area, i.e., Zhejiang province, from 1990 to 2020, using a five-year interval, i.e., the years 1990, 1995, 2000, 2005, 2010, 2015, and 2020. These periods were used in conducting the spatial autocorrelation. The specific results of the global spatial autocorrelation are presented in Table 2 using Moran’s I index of urban expansion.

As presented above, the results of all the time nodes under consideration (i.e., the years 1990, 1995, 2000, 2005, 2010, 2015, and 2020) passed the significance test. The result indicated a positive Moran’s I index that gradually increased from 0.3246 in 1990 to 0.4050 in 2005. The index further rose to 0.4335 in 2020. This outcome suggests that the urban land use expansion in each district and county of Zhejiang province exhibited a significant positive autocorrelation that steadily increased over the period between 1990 and 2020. Therefore, it is imperative to establish a spatial econometric regression model for further analysis of the study areas’ results.

#### 4.1.2. Local Spatial Autocorrelation Result

**Figure 3:**
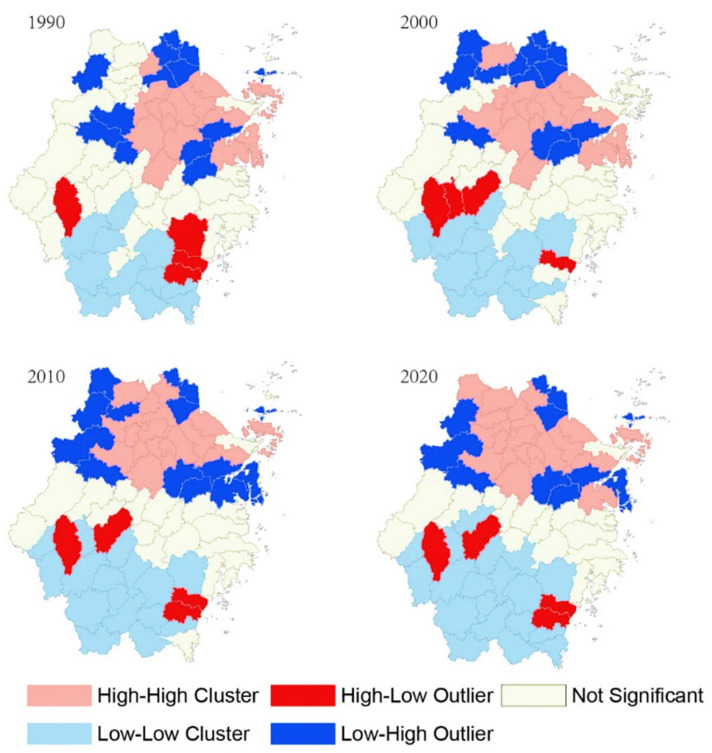
Mapping of local spatial autocorrelation of urban expansion from 1990 to 2020 (*p* < 0.05).

The locations of the spatial clusters and outliers mapped after performing the local spatial autocorrelation tests in the study area from 1990 to 2022 are presented in Figure 3.

The “high-high cluster” areas of urban development were mainly concentrated in the northern region of Zhejiang province, comprising cities that included Hangzhou, Ningbo, Shaoxing, Jiaxing, and Huzhou. These areas have flat terrain and dense river networks. The high-high cluster region of Zhejiang province is one of the most economically developed areas in China and has continuously attracted a large populace due to the socio-economic development of cities within the region. In contrast, the “low-low cluster” areas were predominantly distributed in the central and southern parts of Zhejiang, comprising cities that included Quzhou, Lishui, and some parts of Wenzhou and Jinhua. These regions are in mountainous areas, having a short supply of urban land use, i.e., inadequate land resources for urban development. A “high-low outlier” was observed in the southern region of the study area’s central urban fabric. This included cities such as Quzhou, Wenzhou, and Jinhua. Compared with other counties under the jurisdiction of these cities, these central urban areas, i.e., the high-low outliers, represented the local political, economic, and cultural hub, which gathered a lot of resources and exhibited a peak during the depression. The results also showed a “low-high outlier”, observed in areas such as the west of Hangzhou, the west of Huzhou, the south of Ningbo, and areas of smaller scale. Such regions have numerous mountains, which means these areas are not suitable for large-scale urban development.

### 4.2. Urban Land Use Expansion in Zhejiang Province from 1990 to 2020

In terms of urban growth and expansion, the built-up areas in Zhejiang Province significantly increased from 1955.9 km^2^ in 1990 to 8855.49 km^2^ in 2020, indicating an increase of 6899.59 km^2^ over the three decades. The result revealed the steady development and expansion of urban areas, which increased from 1.9% to 8.59% between 1990 and 2020. This development led to a decline in agricultural and cultivated land areas, which constantly decreased from 28,660.55 km^2^ in 1990 to 24,339.87 km^2^ in 2020, signifying an agricultural land decline of approximately 4.19% between 1990 and 2020.

**Figure 4:**
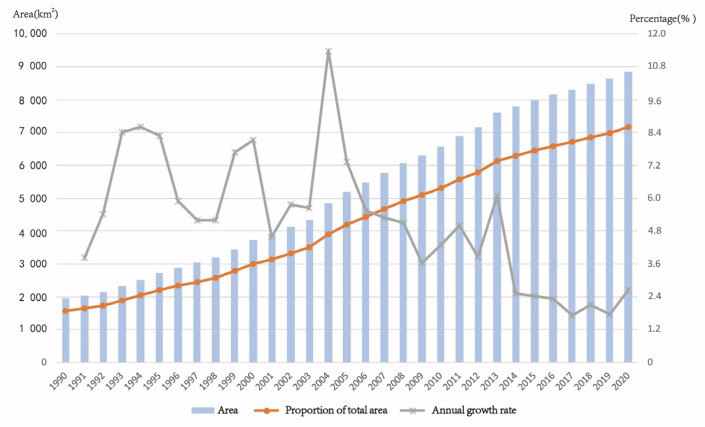
Change in built-up areas from 1990 to 2020. Note: The annual growth rate is the built-up land area of each year minus the preceding year and then divided by the built-up land area of the preceding year.

The annual growth rate of the study area, i.e., Zhejiang Province, fluctuated throughout the study period. Figure 4 indicates a dramatic trend in the urban growth rate, with a gradual increase and decrease during the different periods between 1990 and 2020. A clear cut-off point was observed in 2004, when the growth rate peaked at 11.38%. Then, it gradually declined and slowed down to its lowest rate in 2019.

### 4.3. Contributions of Other Land Uses to Built-Up Area Expansion

The land use transfer matrixes of the study area, i.e., Zhejiang Province, between 1990 and 2020 are presented in Table 3, Table 4, Table 5 and Table 6. The results show the changes in land uses of each LULC class over the 30 years of the study period.

The analysis of the transformation of built-up areas in the study area indicated rapid, continuous, and progressive urbanization due to the economic development of the province. The total contribution of other LULC classes to built-up area expansion was approximately 7009.75 km^2^. The study results revealed agricultural farmland to be the largest contributor, i.e., inflow source, for built-up areas, contributing a total land mass of about 6091.78 km^2^, accounting for about 86.9%, whereas forest land and water bodies contributed 6.94% and 6.06%, respectively, to the expansion of urban areas in Zhejiang Province over the period between 1990 and 2020. These three LULC classes, i.e., agricultural land, forest areas, and water bodies, accounted for 99.9% of the study area’s total urban land use expansion. The study also revealed the outflow of built-up areas to other LULC classes, amounting to approximately 110.16 km^2^. The outflow contributed to the study area’s farmlands and water bodies, suggesting the implementation of activities that included land reclamation and the construction of water conservation facilities.

**Figure 5:**
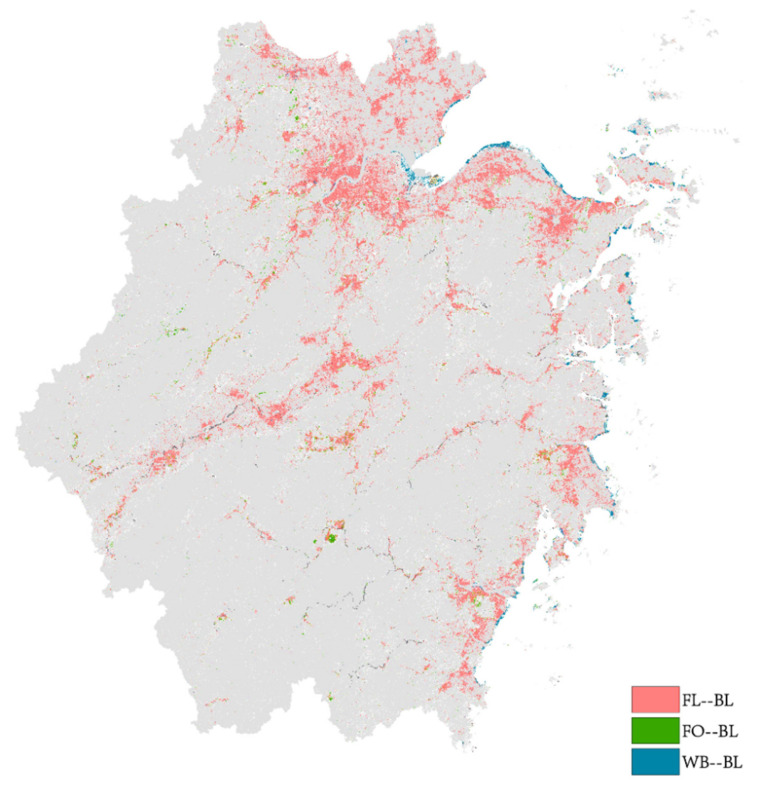
Spatial mapping of the transformation of agricultural areas, forestland, and water bodies into built-up areas from 1990 to 2020.

We further analyzed the spatio-temporal differences in the inflow and outflow of built-up areas during different study periods. From 1990 to 2000, 1620.21 km^2^ of farmland was transformed into built-up areas. The total area rose further to 2598.67 km^2^ from 2000 to 2010 and declined to 2003.43 km^2^ from 2010 to 2020. This decline in the last period could be attributed to the resolutions of the 18th National Congress of the Communist Party of China in 2012, which developed sustainable policies to change the country’s economic development plan. The policies emphasized environmental protection, food security, and farmland preservation. Since then, the country’s provinces have implemented more resilient measures to protect agricultural lands. The land use transfer matrix results also revealed a similar transformation of forestland and water bodies into built-up areas due to the numerous alterations observed during the different study periods. Furthermore, a significantly higher land use alteration was observed from 2000 to 2010 than from 1990 to 2000 and 2010 to 2020. The spatial mapping of the transformation of farmland, forestland, and water bodies into built-up areas throughout the period between 1990 and 2020 is shown in Figure 5.

The alteration of farmland into built-up areas mainly occurred in the northern region of Zhejiang province, i.e., the Hangjiahu region; the Central Zhejiang basin, i.e., the Jinhua region; the coastal alluvial plain formed by Yongjiang, i.e., Ningbo; Jiaojiang, i.e., Taizhou; and Oujiang, i.e., Wenzhou in the east. The features and characteristics of these areas include flat terrain, convenient transportation, and a developed economy, which are the main factors that promote urbanization and urban development in Zhejiang Province. The transformation of water bodies into built-up areas was observed on the eastern coast of the study area, which is attributed to the land reclamation activities of such places. Such activity has led to severe challenges in managing the coastal ecological environment. Thus, the government of the Zhejiang province in 2019 enforced laws that require all localities to fully implement the policy of the strict management and control of reclamation. In addition, the enforcement authorities banned all new reclamation projects in the province except for major national strategic projects.

### 4.4. Spatial Distribution of Built-Up Area Development

**Figure 6:**
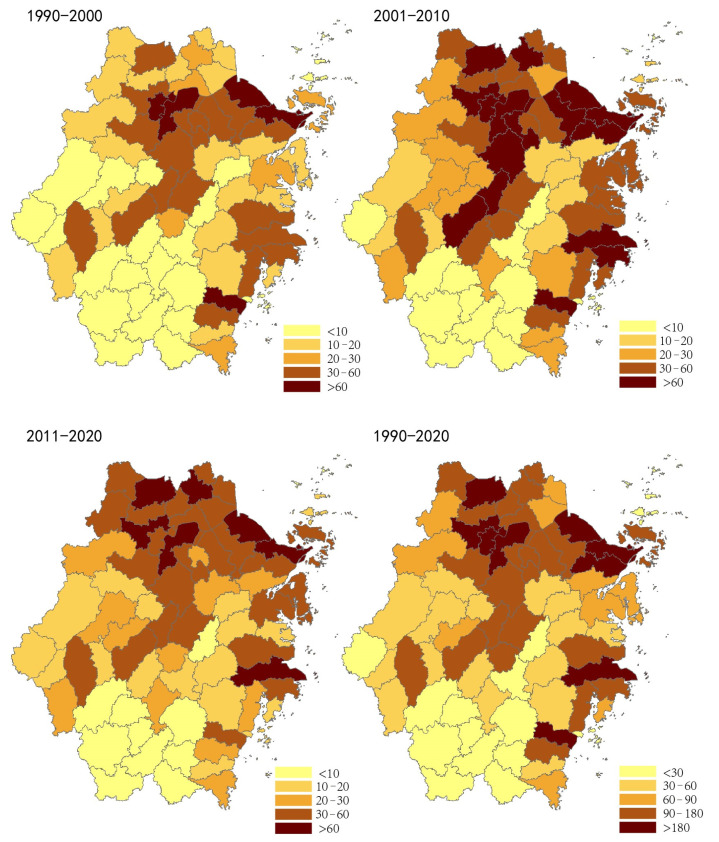
The built-up area expansion of counties in Zhejiang Province from 1990 to 2020 (km^2^).

In this section, we compared and analyzed the built-up area expansion of different counties in Zhejiang Province during the different study periods under consideration. The built-up area expansion refers to the difference in area of built-up land between two periods. It is computed as area in time 2 minus areas in time 1. The spatial distributions mapped and presented in Figure 6 indicate 22 counties in the study area having an built-up land growth of less than 10 km^2^ during period 1 (i.e., 1990–2000), 15 during period 2 (i.e., 2000–2010), and 13 during period 3 (i.e., 2010–2020), which were mainly concentrated areas such as Jinhua, Quzhou, and Lishui.

Throughout the three periods, there were 13 counties in Zhejiang Province that consistently exhibited an urban expansion less than 10 km^2^. These counties were Songyang, Suichang, Qingyuan, Jingning, Longquan, Qingtian, Yunhe, Wencheng, Taishun, Panan, Dongtou, Daishan, and Shengsi. The results also revealed three counties in the above areas to be located on islands, whereas ten counties were in mountainous areas. Land resources in such islands and mountainous regions were in short supply. In addition, the economic development of these areas was relatively slow, severely affecting the scale of urban growth and expansion.

**Figure 7:**
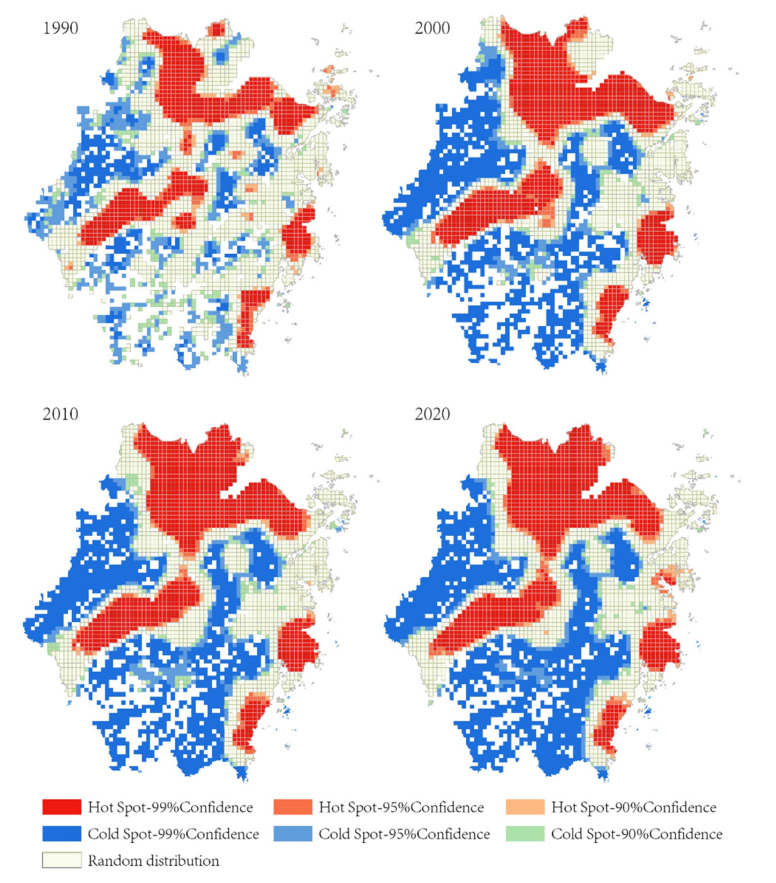
Evolution of urban development hotspots in Zhejiang Province from 1990 to 2020 (5 km × 5 km grid).

During the study period from 1990 to 2000, the result indicated five counties of the study area in which the built-up areas expanded by more than 60 km^2^. These areas were mainly located in the northern plain and the eastern coastal plain of Zhejiang province. The study results showed that three core areas of the province, i.e., the Hangzhou, Ningbo, and Wenzhou urban areas, exhibited rapid urban development radiating to the neighboring areas. Between 2000 and 2010, the number of counties with urban growth greater than 60 km^2^ increased to 16, with such areas further spreading to the Taizhou coastal plain and the central basin of Zhejiang. These areas gradually expanded and amalgamated with the growth trends of Hangzhou, Ningbo, Jinhua, Wenzhou, and Taizhou as the core. In the decade between 2010 and 2020, the study results indicated that seven counties of the study area had more than 60 km^2^. These areas were mainly located in the northern plain of Zhejiang province and the Ningbo and Taizhou coastal areas. Figure 7 presents the spatial mapping of the rapid urban expansion and development of Zhejiang province during the study period between 1990 and 2020.

The spatial distribution result revealed the following.

(1) The Northern plain of Zhejiang province accommodated the urban area of Hangzhou as the central hub of rapid urban expansion in the study area. Such growth and development expanded to the surrounding areas of Xiaoshan, Yuhang, Deqing, Huzhou, Haining, Jiaxing, Fuyang, Shaoxing, and Zhuji.

(2) The urban area of Ningbo was the center of rapid urbanization that spread to the surrounding urban areas of Cixi, Yuyao, and Zhoushan, especially Cixi, in which the built-up areas increased by more than 60 km^2^ over the three periods under study.

(3) The results for the central basin of Zhejiang province indicated that Jinhua’s urban area was the center of rapid expansion. This area expanded relatively to the surrounding areas of Yiwu, Dongyang, and Yongkang.

(4) The coastal areas of Zhejiang, Wenzhou, and Taizhou also exhibited rapid urban development, with the neighboring coastal areas of Wenling, Linhai, Yueqing, and Ruian revealing a similar trend of expansion.

The growth and expansion of these cities and counties suggest that the study area, i.e., Zhejiang Province, has gradually acquired the development mode of a metropolitan circle, forming four urban agglomeration areas. These areas include Hangzhou, Ningbo, Jinhua–Yiwu, and Wenzhou–Taizhou.

## 5. Analysis of the Drivers/Influencing Factors of Built-Up Area Expansion

### 5.1. Selection of Regression Models

In order to remedy the influence of different data dimensions and reduce heteroscedasticity in this study, natural logarithms were taken as variables simultaneously, and the maximum likelihood estimation (MLE) method was used for regression analysis.

To choose the most suitable and appropriate spatial econometric model, it is often necessary to combine practical problems using a series of relevant model tests (Table 7). To achieve this, the LM test and the robust LM test were performed in this study. We found that the results of the two tests both passed the 1% significance level, suggesting the simultaneous existence of the spatial lag model and the spatial error model. Therefore, the spatial Durbin model was the preferred model based on studies of a similar nature [67,68]. Then, the Wald test was further conducted in this study on the spatial Durbin model in order to determine whether it could be simplified to the spatial lag model or the spatial error model. The test results led us to reject the null hypothesis that the spatial Durbin model could be simplified into a spatial lag model or a spatial error model with a significance level of 10%. Hence, the spatial Durbin model was chosen for this study. Due to the utilization of panel data for counties and cities in Zhejiang Province over the last three decades, regional heterogeneity resulting from spatial geographic locations and the impact of policy changes was inevitable. Theoretically, the fixed-effect model was more suitable than the random-effect model. Furthermore, the results of the Hausman test conducted for the fixed effect model and the random effects model showed that the fixed-effect model was more appropriate. Hence, the fixed effect space Durbin model was finally adopted in this study.

In consideration of the robustness and reliability of the results, we conducted regression analysis on SLM, SEM, and SDM at the same time using two different spatial weights. The results are shown in Table 8. The calculation results showed that although the coefficients of the estimated results were different, their signs and significance did not fundamentally change. This also indicates that the results in Table 8 are robust and reliable.

### 5.2. Analysis of Spatial Effects

In the two regression scenarios of the spatial Durbin model, the spatial autoregressive coefficient ρ passed the significance test with both weights, i.e., $W_{1}$ and $W_{2},$ using a 1% significance level benchmark. This result signifies that the growth and expansion of the built-up areas in neighboring areas had a positive spillover effect on the development of the local built-up areas. These findings are consistent with the studies by He et al. [54] and Deng et al. [55], which highlighted similar scenarios in the outcomes of their research. The positive spillover effect could be attributed to the strategic urban development and follow-up assessments conducted by local government authorities. Such areas have contributed immensely to the GDP of China through the provision of development land and their capacity to serve as the country’s major contributor to socio-economic development [42]. We evaluated the fitting effect of the spatial Durbin model by integrating the R-squared and logLik indices. The model with the best explanatory power had a higher R-squared value and a smaller absolute value of logLik. Therefore, we utilized the queen contiguity weight $W_{1}$ for further analysis of the results.

We focused on the marginal contribution of variables because the coefficient of the explanatory variable and the coefficient of the spatial lag term in the regression results of the spatial Durbin model could not directly represent the magnitude of this contribution. Therefore, the traditional method of explaining the coefficient of the non-spatial model cannot be quantitatively described. We further employed the method of partial differential decomposition to translate the variables presented in Table 8 into the direct and indirect effects highlighted in Table 9. The direct effect is the local effect, which represents the influence of the changes of local explanatory variables on the growth and expansion of local built-up areas, whereas the indirect effect is the spatial spillover effect, which represents the influence of local explanatory variables on the growth and expansion of built-up land in neighboring areas. The total effect is based on the sum of direct and indirect effects.

Based on the various results regarding the total effects, socio-economic development had the most significant influence on the growth and expansion of built-up areas. The regression results showed that urban land use—i.e., built-up areas—increased by 0.8428% for every 1% increase in GDP, followed by population and fiscal expenditure, which had marginal effects of 0.1481% and 0.1230%, respectively.

From the perspective of the decomposition of effects, population growth contributed significantly to the local expansion of built-up areas, which inhibited the expansion of neighboring areas. The results indicated direct and indirect marginal effects of 0.4999% and −0.3517%, respectively. The increase in the urban population not only led to an increase in housing demand but also led to the quest for better employment, education, medical, and other infrastructural and social amenities, resulting in the massive growth and expansion of urban areas through the development of built-up areas. However, the results also indicated a “siphoning effect” on the populations between neighboring regions. Central urban areas with a conducive environment and high-quality public service further attracted a large populace from neighboring small and medium-sized cities. This development slowed down the urban development of such neighboring cities and counties.

Regional GDP also significantly promoted the expansion of built-up areas in both local and neighboring areas. The study results revealed that for every 1% increase in regional GDP, this led to increases in built-up area of 0.3907% and 0.4521% in the local and neighboring areas of the study area, respectively. This indicates that rapid economic growth produces certain positive externalities and drives the rapid economic development of the surrounding areas.

In addition, the increase in the coefficient of the industrial structure exhibited a significant inhibitory effect on the expansion of built-up areas in Zhejiang province. However, the results suggested that this had no significant effect on the neighboring areas. As the coefficient of the industrial structure increased by 1%, the built-up area decreased by 0.1646%. Pandey et al. [69], Gao et al. [60], and Wu et al. [70] indicated that the land use efficiency of service industries (such as finance, education, cultural industries, etc.) is much higher compared with labor-intensive industries. Therefore, its prosperity and development have slowed the transformation of farmland into built-up areas.

The increase in foreign direct investment also exhibited a significant effect on the expansion of built-up areas, while exhibiting some inhibiting effect on the expansion of neighboring urban areas. The study results indicated that for every 1% increase in foreign direct investment, the local built-up area expanded by 0.0234%, whereas neighboring urban areas decreased by 0.0356%. The implementation of new policies related to China’s economic development and the continuous influx of foreign capital has led to a substantial demand for land resources in most urban areas. Such land is used for industrial, infrastructure, and real estate investments. Over the years, this development contributed to neighboring regions having a competitive effect on investment [43].

Furthermore, the increase in fixed-asset investment also demonstrated a significant effect on the expansion of built-up areas in the most urban areas of cities, whereas a significant inhibiting effect was observed on the expansion of neighboring areas. For every 1% increase in fixed-asset investment, the local built-up area increased by 0.1063%, whereas the neighboring built-up area decreased by 0.2510%. The influence of fixed asset-investment was similar to that of foreign direct investment, suggesting a competitive effect between local and neighboring areas.

Finally, increased fiscal expenditure showed no significant effect on the expansion of local built-up areas. However, it revealed a significant promoting effect on the expansion of built-up areas in neighboring counties and cities. The results indicated that a 1% increase in fiscal expenditure led to a 0.1391% increase in the built-up areas of neighboring areas. The possible reason for this is the strategic follow-up among local governments, which further promotes the expansion of built-up land through active fiscal policies.

### 5.3. Further Analysis of Different Study Periods

The research was divided into three (3) study periods: period one, i.e., 1990–2000; period two, i.e., 2000–2010; and period three, i.e., 2010–2020. The three periods were used to analyze urban land use changes and the various factors influencing the growth and expansion of built-up areas in each county of Zhejiang Province. The regression results of the three periods are presented in Table 10.

Based on the direct effect results, the influence of population growth on the expansion of built-up areas in Zhejiang province declined by 0.5581%,0.5732%, and 0.3202% during period 1, i.e., 1990–2000; period 2, i.e., 2000–2010; and period 3, i.e., 2010–2020, respectively. However, the influence of regional GDP on the expansion of built-up areas increased from 0.3328% to 0.5697% from periods 1 to 3. This increase reflects the influence of regional gross domestic product on the urban development of Zhejiang during the different periods from 1990 to 2020. Furthermore, the influence of industrial structure increased from −0.0886% to −0.2291% between periods 1 and 3, indicating the intensive utilization of land for various industrial development purposes. The influence of foreign direct investment exhibited a slight decrease from 0.0312% to 0.0235%. This decline became insignificant during period 3, suggesting its weak influence on the expansion of built-up areas.

On the contrary, investment in fixed assets had an increasing influence on the expansion of built areas in Zhejiang province, from an insignificant influence at the early stage to 0.1680% and later to 0.2092%. The study results revealed the variation in local financial expenditure as the most significant factor, having an influence of 0.1377% in period one and −0.1998% in period three. This difference suggests that local financial expenditure had a strong positive effect on the expansion of built-up areas during the period between 1990 and 2000. Local financial expenditure constantly contributed to the extensive development of urban areas through the expansion of urban infrastructures and social amenities [71]. Still, this impact weakened and turned into an inhabiting effect over the years.

As for the indirect effect, the study results indicated that the population coefficient during period one was −0.4612%, whereas the coefficient of GDP was 0.4583%. Population growth and GDP coefficient had a negative and positive influence on the growth and expansion of built-up areas in local and neighboring areas of Zhejiang province during the whole period under consideration. Both showed an initial increasing trend and then a decreasing trend in the subsequent two periods. The results obtained during period 2 were −0.4828% and −0.2187% and during period 3 they were 0.5724% and 0.3063%, respectively. These changes in the influence of fixed-asset investment are similar to those of population growth and gross regional product, whereas foreign direct investment had the opposite outcome, showing a negative influence on the expansion of urban areas.

### 5.4. Further Analysis of Different Regions

In this section, we divide the study area into mountain and non-mountain counties. According to the official division of China, there are 26 mountain counties, of which the economic development levels are relatively outdated in relation to Zhejiang Province. Therefore, due to these insufficient data, we sought to analyze the various drivers/influencing factors of urban land use expansion in different regions of Zhejiang Province over the past 30 years.

Based on the regression results presented in Table 11, the study results showed remarkable differences in the various drivers/factors influencing the expansion of built-up area in the mountainous and non-mountainous areas of Zhejiang province, especially in relation to the population of the study area. The results indicate that the direct, indirect, and total effects of population in the mountain counties of the study area were significantly negative. This result suggests that growth in the population of mountain counties will inhibit the expansion of built-up areas. The population in the mountain counties of Zhejiang province did not increase as much as the urban areas increased over the three decades of the study period. After comparing the population data from 1990 to 2020, we found that the population of 26 counties in the mountain areas of the study area increased by only 325,500 in 30 years, with most of the counties in this area showing population losses. However, the populations of non-mountain counties during the same period exhibited a constant inflow, with a population increase of 22 million. This, on the other hand, also explains the underlying reason for the indirect effect of population observed in non-mountain counties, which was significantly positive throughout the study period.

The study revealed different leading factors contributing to built-up area expansion in mountainous and non-mountainous areas. In the mountainous areas, the total effect of GDP was the largest, at 2.8248%, whereas the total effect of the population was −2.4441%. The result suggests that the expansion of the built-up area in mountainous counties was facilitated by socio-economic development. For non-mountainous counties, the total effect of GDP was insignificant, whereas the total effect of population was 0.7855%. This result suggests that built-up area expansion in non-mountainous counties was mainly facilitated by population growth.

## 6. Conclusions

In this paper, we employed remote sensing data and GIS, using spatial econometrics, R language, and other innovative methods to analyze the spatial characteristics, nature of expansion, and various factors influencing the changing dynamics of built-up areas in Zhejiang Province from 1990 to 2020. We found that spatial effects were very important in the geographical process, and ignoring spatial effects may lead to deviations in results. That is to say, in the process of studying urban land expansion, we should fully consider the spatial spillover of the elements of neighboring administrative regions. We can summarize the major findings of this study as follows.

Firstly, the built-up area of Zhejiang Province increased significantly from 1955.9 km^2^ to 8855.49 km^2^ from 1990 to 2020, with its total landmass increasing from 1.9% in 1990 to 8.59% in 2020. In contrast, the area of agricultural farmland decreased rapidly from 28,660.55 km^2^ in 1990 to 24,339.87 km^2^ in 2020. This decline accounted for the 4.19% agricultural land decline between 1990 and 2020.

Secondly, agricultural farmland was observed to be the largest inflow, i.e., the largest land source for the expansion of built-up areas in Zhejiang Province during the study period from 1990 to 2020, with the transformation of 6091.78 km^2^ of farmlands into built-up areas, accounting for 86.9% of the total built-up area expansion. Forestland and water bodies contributed 486.62 km^2^ and 424.8 km^2^, respectively, to the growth of built-up areas, accounting for 6.94%, and 6.06%, respectively. These three LULC classes accounted for 99.9% of the total inflow to built-up area land.

Thirdly, the built-up areas of Zhejiang province showed significant spatial clustering characteristics, with the “high-high cluster” mainly concentrated in the North of Zhejiang Province, whereas the “low-low clusters” were located primarily in the south of the province. The expansion hotspots were distributed in the urban areas of Hangzhou, Ningbo, Jinhua, and Wenzhou–Taizhou, which suggested the gradual development of the urban character of the province, with Hangzhou, Ningbo, Jinhua–Yiwu, and Wenzhou–Taizhou forming the respective centers.

Fourthly, the growth and development of built-up areas in neighboring areas indicated a positive spillover effect on local areas. The study area’s population growth, economic development, fixed-asset investment, and foreign direct investment had significant roles in promoting the expansion of local built-up areas, whereas industrial structure exhibited an inhibitory role. For urban expansion in neighboring areas, economic development and industrial structure showed a significant positive spatial spillover effect, whereas population growth, fixed-asset investment, and foreign direct investment demonstrated significant negative spatial spillover effects.

Finally, economic development served as the main factor influencing the expansion of built-up areas during the different study periods under consideration. However, this impact was weakened over the years, with the restraining effect of the study area’s industrial restructure gradually increasing. The study results also indicated the influence of economic development on the built-up area expansion of the different regions in Zhejiang province. Economic development had a stronger influence on the expansion of the built-up areas in mountainous counties than in non-mountainous counties, which may contribute to compensating for the negative effect of population losses in mountainous counties on built-up area expansion.

However, in our study we did not consider the influence of external factors on the expansion of built-up areas in the inter-provincial border areas, especially in the northern Zhejiang plain, where neighboring Jiangsu and Shanghai are located. Furthermore, a geographically weighted regression model integrated with spatial panel data should be embraced in future studies of a similar nature in order to analyze the spatial heterogeneity of Zhejiang Province.

## 7. Recommendations

In the three decades of the study period, rapid urbanization not only provided a strong impetus for the socio-economic development of Zhejiang Province, but also occupied a large amount of farmland, causing a series of ecological and environmental problems, which had a noticeable impact on the sustainable socio-economic development of urban and rural areas in the future. Therefore, the effective scientific identification of the characteristics and laws of the spatial and temporal patterns of evolution of construction land is crucial in order to reasonably predict the future spatial patterns of urbanization and to promote regional sustainable development.

Based on the study’s results, we put forward the following policy recommendations, which are not only for Zhejiang Province, but which may also be useful for China and other regions of the world that are experiencing rapid urbanization.

(i) The expansion of urban construction land is inevitable and important. We oppose inefficient and disorderly land development, but we also support the further expansion of construction land in some areas that already have the advantage of urbanization. For example, core big cities should play a leading role in economic development and become key development zones. However, in places far away from big cities and with remote physical and geographical conditions, especially in mountainous areas, more ecological protection functions should be employed. In this way, sustainable development can be achieved in a wider range.
(ii) Policymakers should break through administrative boundaries and coordinate regional development from a higher and more comprehensive perspective. Taking Zhejiang Province as an example, priority should be given to the development of the four metropolitan areas in Zhejiang Province, breaking through the restrictions of administrative barriers regarding the flow of economic resource elements, optimizing the urban spatial system, and realizing the coordinated development of large, medium, and small cities.
(iii) The transformation and upgrading of the industrial structure should be accelerated. Developing tertiary industry can also be seen as an effective way to ease the conflict between cultivated land and construction land in developing countries. Our analysis indicates the positive effect of the development of the tertiary industry on improving land use efficiency and thus slowing down the occupation of farmland in Zhejiang. Therefore, we should vigorously promote the transformation of traditional industries into ecological, scientific, and service industries.

## Figures and Tables

**Table 1:** Drivers and factors influencing urban expansion.

Variable	Description	Attribute
BUA	Area of built-up land in the region at year’s end	Explained variable
RTP	Region’s total population at year’s end	Explanatory variable
GDP	Year-end gross regional product	Explanatory variable
STR	The ratio of the value added of the tertiary industry to the secondary industry in that year	Controlled variable
FDI	Total foreign direct investment that year	Controlled variable
INV	Total investment in fixed assets for that year	Controlled variable
LFE	Total local government expenditure for that year	Controlled variable

**Table 2:** Moran’s I index of urban expansion of counties in Zhejiang Province (1990–2020).

S/No	Year	Moran’s I	Z-Score	*p*-Value
1.	1990	0.3246	4.1720	0.000
2.	1995	0.3336	4.2903	0.000
3.	2000	0.3572	4.5807	0.000
4.	2005	0.4050	5.1487	0.000
5.	2010	0.4233	5.3735	0.000
6.	2015	0.4312	5.4608	0.000
7.	2020	0.4335	5.4827	0.000

**Table 3:** Transfer matrix of land use change in Zhejiang Province from 1990 to 2020 (km^2^).

1990 2020	BL	FL	FO	GR	WB	BS	Inflow
**BL**	1845.74	6091.78	486.62	6.27	424.8	0.28	7009.75
**FL**	25.70	20,350.41	3547.44	8.08	408.22	0.02	3989.46
**FO**	0.39	1502.87	65,303.64	14.18	59.91	0	1577.35
**GR**	0.00	1.60	3.68	3.97	0.19	0	5.47
**WB**	84.00	713.23	24.64	0.75	2206.76	0	822.62
**BS**	0.07	0.66	0.2	0.04	0.79	0.01	1.76
**Outflow**	110.16	8310.14	4062.58	29.32	893.91	0.3	-

**Table 4:** Transfer matrix of land use change in Zhejiang Province from 1990 to 2000 (km^2^).

1990 2000	BL	FL	FO	GR	WB	BS	Inflow
**BL**	1901.57	1620.21	57.96	3.83	139.01	0.14	1821.15
**FL**	1.91	25,033.72	1285.4	3.4	184.68	0.03	1475.42
**FO**	0.13	1672.49	68,019.22	11.6	43.15	0	1727.37
**GR**	0	2.02	0.44	14.11	0.11	0	2.57
**WB**	52.29	332.1	3.2	0.31	2733.68	0.02	387.92
**BS**	0	0.01	0	0.04	0.04	0.12	0.09
**Outflow**	54.33	3626.83	1347	19.18	366.99	0.19	-

**Table 5:** Transfer matrix of land use change in Zhejiang Province from 2000 to 2010 (km^2^).

2000 2010	BL	FL	FO	GR	WB	BS	Inflow
**BL**	3593.42	2598.67	177.69	1.34	193.37	0.1	2971.17
**FL**	9.42	21,768.83	1502.29	2.94	223.63	0	1738.28
**FO**	0.15	1427.19	68,054.95	2.96	27.46	0	1457.76
**GR**	0.02	17.86	1.22	9	0.74	0.02	19.86
**WB**	119.71	696.33	10.41	0.34	2675.75	0	826.79
**BS**	0	0.26	0.03	0.1	0.65	0.09	1.04
**Outflow**	129.3	4740.31	1691.64	7.68	445.85	0.12	-

**Table 6:** Transfer matrix of land use change in Zhejiang Province from 2010 to 2020 (km^2^).

2010 2020	BL	FL	FO	GR	WB	BS	Inflow
**BL**	6514.28	2003.43	153.2	17.34	166.44	0.8	2341.21
**FL**	6.9	20,676.76	3109.57	3.56	543.02	0.06	3663.11
**FO**	0.07	631.91	66,244.91	1.97	2.13	0	636.08
**GR**	0	1.81	1.85	5.64	0.1	0.04	3.8
**WB**	43.33	193.1	3.18	0.07	2789.7	0	239.68
**BS**	0.01	0.1	0	0.28	1.15	0.23	1.54
**Outflow**	50.31	2830.35	3267.8	23.22	712.84	0.9	-

**Table 7:** Test results of the spatial panel model selection process.

S/No	Test	Statistic	*p*-Value
1.	LM-lag	137.18	0.000
2.	LM-error	119.55	0.000
3.	RLM-lag	26.97	0.000
4.	RLM-error	9.35	0.002
5.	Wald spatial lag	12.42	0.053
6.	Wald spatial error	19.09	0.004
7.	Hausman	890.4	0.000

**Table 8:** Regression results of SLM, SEM, and SDM.

Variables	SLM		SEM		SDM	
	$W_{1}$	$W_{2}$	$W_{1}$	$W_{2}$	$W_{1}$	$W_{2}$
ln RTP	0.4707 ***	0.4652 ***	0.4911 ***	0.4759 ***	0.5134 ***	0.4749 ***
	(19.8720)	(19.6014)	(20.7455)	(20.1474)	(21.5537)	(20.3221)
ln GDP	0.4264 ***	0.4482 ***	0.3979 ***	0.4259 ***	0.3733 ***	0.4356 ***
	(13.3723)	(14.1929)	(12.5133)	(13.4061)	(11.6445)	(13.7442)
ln STR	−0.1514 ***	−0.1498 ***	−0.1523 ***	−0.1442 ***	−0.1627 ***	−0.1497 ***
	(−6.2092)	(−6.1210)	(−6.3748)	(−5.9100)	(−6.8745)	(−6.2418)
ln FDI	0.0175 ***	0.0184 ***	0.0251 ***	0.0215 ***	0.0248 ***	0.0239 ***
	(5.0767)	(5.3312)	(7.3162)	(6.2406)	(7.2353)	(6.9654)
ln INV	0.0822 ***	0.0753 ***	0.1071 ***	0.0957 ***	0.1159 ***	0.0939 ***
	(3.6273)	(3.3155)	(4.7723)	(4.2127)	(5.1904)	(4.1643)
ln LFE	0.0017 *	−0.0059	−0.0249	−0.0195	−0.0214	−0.0382
	(0.0614)	(−0.2155)	(−0.9122)	(−0.7111)	(−0.7936)	(−1.4072)
ρ	0.0450 ***	0.0038			0.1825 ***	0.0899 ***
	(3.4814)	(0.2698)			(6.4360)	(2.9544)
λ			0.2342 ***	0.1242 ***		
			(8.3384)	(4.0201)		
W˟ ln RTP					−0.3923 ***	−0.0999 *
					(−7.6983)	(−1.8725)
W˟ ln GDP					0.3158 ***	0.1323 **
					(5.3028)	(1.9894)
W˟ ln STR					−0.0122	−0.2747 ***
					(−0.2362)	(−4.3859)
W˟ ln FDI					−0.0347 ***	−0.0361 ***
					(−6.5144)	(−6.0072)
W˟ ln INV					−0.2343 ***	−0.3049 ***
					(−5.6002)	(−7.2419)
W˟ ln LFE					0.1220 ***	0.2691 ***
					(2.7406)	(5.4943)
R-squared	0.8975	0.8969	0.8965	0.8968	0.9053	0.9033
logLik	−580.02	−586.07	−579.3	−583.1	−508.75	−522.90

Note: ***, **, and * indicate that the results are significant at the 0.01, 0.05, and 0.1 levels, respectively, with T-statistics in parentheses.

**Table 9:** Statistical data of the direct, indirect, and total effects of explanatory variables.

Variables	Effect	$W_{1}$	$W_{2}$
**RTP**	*Direct effect*	*0.4999* ***	*0.4738* ***
	*Indirect effect*	*−0.3517* ***	*−0.0616*
	Total	0.1481 **	0.4121 ***
**GDP**	*Direct effect*	*0.3907* ***	*0.4392* ***
	*Indirect effect*	*0.4521* ***	0.1848 **
	Total	0.8428 ***	0.6240 ***
**STR**	*Direct effect*	*−0.1646* ***	*−0.1558* ***
	*Indirect effect*	*−0.0493*	*−0.3105* ***
	Total	−0.2139 ***	−0.4663 ***
**FDI**	*Direct effect*	*0.0234* ***	*0.0232* ***
	*Indirect effect*	*−0.0356* ***	*−0.0366* ***
	Total	−0.0122 *	−0.0134 *
**INV**	*Direct effect*	*0.1063* ***	*0.0877* ***
	*Indirect effect*	*−0.2510* ***	*−0.3195* ***
	Total	−0.1447 ***	−0.2318 ***
**LFE**	*Direct effect*	*−0.0160*	*−0.0326*
	*Indirect effect*	*0.1391* ***	*0.2863* ***
	Total	0.1230 **	0.2537 ***

Note: ***, **, and * indicate that the results are significant at the 0.01, 0.05, and 0.1 levels, respectively.

**Table 10:** Statistical comparison of the regression results obtained during periods 1, 2, and 3.

Impact	Variables	Period 1		Period 2		Period 3	
**Direct**	RTP	0.5581 ***	(9.8126)	0.5732 ***	(14.1177)	0.3202 ***	(9.4878)
	GDP	0.3328 ***	(4.7707)	0.3273 ***	(5.7846)	0.5697 ***	(11.8805)
	STR	−0.0886 *	(−1.8059)	−0.2198 ***	(−4.6461)	−0.2291 ***	(−6.0449)
	FDI	0.0312 ***	(4.1615)	0.0235 ***	(3.8268)	0.0056	(1.2303)
	INV	−0.0101	(−0.2292)	0.1680 ***	(4.0663)	0.2092 ***	(5.3840)
	LFE	0.1377 **	(2.4588)	−0.0142	(−0.2812)	−0.1998 ***	(−5.1106)
**Indirect**	RTP	−0.4612 ***	(−3.9602)	−0.4828 ***	(−5.0578)	−0.2187 ***	(−2.8069)
	GDP	0.4583 ***	(3.6465)	0.5724 ***	(4.9238)	0.3063 ***	(2.7415)
	STR	0.0127	(0.1189)	−0.0486	(−0.4587)	−0.1614 *	(−1.7700)
	FDI	−0.0339 ***	(−2.8280)	−0.0218 **	(−2.2731)	−0.0427 ***	(−5.2393)
	INV	−0.2090 **	(−2.5069)	−0.3233 ***	(−3.7662)	−0.1685 **	(−1.9811)
	LFE	0.2122 **	(2.3341)	0.0915	(1.1271)	0.0939	1.2367
**Total**	RTP	0.0969	(0.7706)	0.0904	(0.8094)	0.1015	(1.1607)
	GDP	0.7911 ***	(5.1467)	0.8996 ***	(6.2795)	0.8760 ***	(6.7652)
	STR	−0.0759	(−0.5903)	−0.2684 **	(−2.0348)	−0.3905 ***	(−3.4963)
	FDI	−0.0028	(−0.2302)	0.0018	(0.1793)	−0.0370 ***	(−3.7839)
	INV	−0.2191 **	(−2.1432)	−0.1554	(−1.4314)	0.0407	(0.3862)
	LFE	0.3499 **	(3.1393)	0.0773	(0.7584)	−0.1058	(−1.2777)

Note: ***, **, and * indicate significance levels of 0.01, 0.05 and 0.1, respectively, with T-statistics in parentheses.

**Table 11:** Comparison of regression results in mountainous and non-mountainous regions.

Effect	Variables	Mountain Counties		Non-Mountain Counties	
**Direct**	RTP	−0.1373 **	(−2.1105)	0.6658 ***	(22.8801)
	GDP	0.9993 ***	(13.3176)	0.1332 ***	(3.3940)
	STR	−0.2450 ***	(−4.8359)	−0.1132 ***	(−4.8835)
	FDI	−0.0031	(−0.8665)	0.0387 ***	(5.6740)
	INV	0.2100 ***	(4.8196)	0.0484 **	(2.1405)
	LFE	−0.1006	(−1.4397)	0.1484 ***	(5.1641)
**Indirect**	RTP	−2.3069 ***	(−13.0832)	0.1196 ***	(2.3751)
	GDP	1.8255 ***	(8.9773)	−0.0133	(−0.2050)
	STR	0.7760 ***	(7.1357)	0.0375	(0.9700)
	FDI	0.0092	(1.1876)	−0.0333 ***	(−3.8902)
	INV	0.1697 *	(1.7918)	−0.0402	(−1.1176)
	LFE	−0.0349	(−0.2186)	0.0397	(0.8658)
**Total**	RTP	−2.4441 ***	(−11.1999)	0.7855 ***	(12.2975)
	GDP	2.8248 ***	(11.3043)	0.1199	(1.4478)
	STR	0.5311 **	(3.9508)	−0.0757 *	(−1.6488)
	FDI	0.0061	(0.6882)	0.0054	(0.5518)
	INV	0.3796 ***	(3.4487)	0.0082	(0.2167)
	LFE	−0.1355	(−0.6738)	0.1881 ***	(4.0529)

Note: ***, **, and * indicate significance levels of 0.01, 0.05, and 0.1, respectively, with T-statistics in parentheses.

## Data Availability

Not applicable.
